# Plaque assay for human coronavirus NL63 using human colon carcinoma cells

**DOI:** 10.1186/1743-422X-5-138

**Published:** 2008-11-12

**Authors:** Petra Herzog, Christian Drosten, Marcel A Müller

**Affiliations:** 1 Bernhard Nocht Institute for Tropical Medicine, Bernhard-Nocht-Str. 74, D-20359 Hamburg, Germany; 2 Institute of Virology, University of Bonn Medical Centre, Sigmund-Freud-Str. 25, 53127 Bonn, Germany; 3 Qiagen Hamburg GmbH, Königstr. 4a, D-22767 Hamburg, Germany

## Abstract

**Background:**

Coronaviruses cause a broad range of diseases in animals and humans. Human coronavirus (hCoV) NL63 is associated with up to 10% of common colds. Viral plaque assays enable the characterization of virus infectivity and allow for purifying virus stock solutions. They are essential for drug screening. Hitherto used cell cultures for hCoV-NL63 show low levels of virus replication and weak and diffuse cytopathogenic effects. It has not yet been possible to establish practicable plaque assays for this important human pathogen.

**Results:**

12 different cell cultures were tested for susceptibility to hCoV-NL63 infection. Human colon carcinoma cells (CaCo-2) replicated virus more than 100 fold more efficiently than commonly used African green monkey kidney cells (LLC-MK2). CaCo-2 cells showed cytopathogenic effects 4 days post infection. Avicel, agarose and carboxymethyl-cellulose overlays proved suitable for plaque assays. Best results were achieved with Avicel, which produced large and clear plaques from the 4^th ^day of infection. The utility of plaque assays with agrose overlay was demonstrated for purifying virus, thereby increasing viral infectivity by 1 log 10 PFU/mL.

**Conclusion:**

CaCo-2 cells support hCoV-NL63 better than LLC-MK2 cells and enable cytopathogenic plaque assays. Avicel overlay is favourable for plaque quantification, and agarose overlay is preferred for plaque purification. HCoV-NL63 virus stock of increased infectivity will be beneficial in antiviral screening, animal modelling of disease, and other experimental tasks.

## Background

Coronaviruses are large enveloped plus-strand RNA viruses that are currently classified in three groups or presumptive genera [1-3]. Group 1 is further divided into two phylogenetic clades exemplified by the transmissible gastroenteritis virus (TGEV) and the porcine epidemic diarrhoea virus (PEDV), respectively. The latter clade contains two prototypic human coronaviruses (hCoV), termed hCoV-229E and -NL63 [4,5]. Like group 1, group 2 contains mammalian CoV. These include two human pathogenic prototypes, termed hCoV-OC43 and -HKU1, several important animal pathogens such as the bovine CoV and the murine hepatitis virus, as well as the SARS-CoV [6-8]. Group 3 contains foremostly avian CoV [9].

HCoV-229E and OC43 as well as the more recently identified hCoV-HKU1 and – NL63 are major causes of common colds in wintertime [10]. HCoV-NL63 was isolated in African green monkey kidney cells (LLC-MK2) from a seven month old infant with bronchiolitis and conjunctivitis [4]. In further investigations the virus was predominantly detected in children with respiratory infections [11-14]. Up to 10% of children with respiratory disease yielded hCoV-NL63 [10,11,15-17].

Because of its relatively high prevalence hCoV-NL63 could become an important model in screening for anti-coronaviral agents [12,18]. Several studies have suggested, e.g., that hCoV protease inhibitors would be cross-reactive among different hCoV [19-21]. Antiviral screening relies on the detection of replicating virus in cell culture. For this and other experimental tasks, plaque assays have proven to be simple in application and efficacious in representing virus viability.

Plaque assays make use of viscous overlays to cover cells immediately after infection, thus limiting virus spread and restricting virus growth to foci of cells at the sites of initial infection. If virus contributes no or low cytopathic effects to cells, these foci may be visualized by immunostaining [22,23]. If virus induces strong cytopathogenic effects (CPE), cells in plaques are lysed and plaques can be visualized by staining of the residual intact cells. Cytopathogenic plaque assays are compatible with high throughput screening [24,25] and facilitate plaque purification and cloning of virus. This in turn is helpful in obtaining virus stocks of optimized infectivity, e.g., by decreasing the amount of defective interfering (di) particles that accumulate during serial passaging of CoV [26].

Important technical achievements have been made in studying NL63 replication, including, most recently, the development of an infectious cDNA clone [27]. Still it is a major obstacle that hCoV-NL63 replicates slowly and at relatively low titres in all current cell cultures, such as LLC-MK2 and Vero-B4 cells [4,28,29]. Because the virus contributes very weak and diffuse CPE to these cells, there is no cytopathic plaque assay available for non-recombinant virus [28].

Although hCoV-NL63 seems to replicate in the upper and lower airways, there are many CoV that predominantly infect the enteric tract, such as TGEV, PEDV, the feline enteric CoV, and the bovine coronavirus [30,31]. SARS-CoV was detected in faecal swabs from SARS patients [32]. SARS-CoV was shown to replicate in colon carcinoma cells (CaCo-2) [33] that are routinely used for growing entero- and adeno-, and astroviruses [34]. Interestingly, SARS-CoV and hCoV-NL63 were shown to use the same receptor for virus entry, the angiotensin converting enzyme 2 (ACE2) [35].

We show here that CaCo-2 cells are highly susceptible for hCoV-NL63 infections and that virus propagation in these cells is much more efficient than in LLC-MK2 cells. By testing different overlays and assay formats we developed cytopathogenic NL63 plaque assays that can be used for analytical and preparative purposes.

## Results and discussion

### Susceptibility of different cell lines to hCoV-NL63 and cytopathogenic effects

LLC-MK2 and Vero cells do not cause clear CPE on infection with hCoV-NL63. Because this virus uses the same receptor as the SARS-CoV, 12 different cell cultures susceptible to SARS-CoV infection were tested for susceptibility to hCoV-NL63 [34,36,37] (Table 1). Cells in six-well plates were infected with 10e4 plaque-forming units of hCoV-NL63 virus stock LLC-MK2 NP. RNA concentrations in supernatants were measured short after virus adsorption (i.e., in fresh medium after washing off of the infection supernatant), and 7 days later (Table 1). Increase of virus RNA was less than 1000-fold in seven of 12 cultures. Interestingly, this included LLC-MK2, the prototype cell culture for NL63. In spite of a low amplification factor these cells showed the usual weak CPE that is typically observed when infected with hCoV-NL63.

**Table 1:** Comparison of hCoV-NL63 replication by real time RT-PCR using different cell cultures

Designation*	**Day 0 [copies/μL]**	**Day 7 [copies/μL]**	**Amplification factor**	**Cytopathogenic effect (CPE)**
Vero E6	6.94e3	3.05e7	4.39e3	None
Vero FM	1.78e4	4.51e9	2.54e5	None
CaCo-2	3.55e3	1.25e10	3.54e6	round and detached, dead cells with cell debris in supernatant, strong effect
Calu1	2.61e4	5.33e6	2.04e2	None
Calu6	7.95e3	5.00e5	6.29e1	None
POEK	8.11e4	3.03e5	3.74e0	None
PK13	2.66e2	7.78e5	2.93e3	None
293lp	3.67e3	3.09e7	8.42e3	None
FeA	1.45e4	5.83e5	4.03e1	None
RD	3.14e5	1.57e4	4.99e-2	None
PS	1.20e4	1.44e6	1.19e2	None
LLC-MK2	4.00e3	2.65e6	6.62e2	round and detached, weak effect

* Vero E6 rhesus kidney cells (ATCC CRL-1586), Vero FM rhesus kidney cells (ATCC CCL-81), CaCo-2 human colon carcinoma (ATCC HTB-37), Calu 1 human lung carcinoma (ICLC HTL95002), Calu 6 human lung carcinoma (ICLC HTL97003), POEK porcine foetal kidney (cell culture collection of the Robert Koch-Institute (RKI), Berlin, Germany), PK13 porcine kidney (cell culture collection of the Bernhard-Nocht-Institute (BNI), Hamburg, Germany), 293 human embryonic kidney (ATCC CRL-1573), FEA feline embryonic fibroblast (kindly provided by Dr. Marcel Asper, NewLab Inc., Cologne), RD human rhabdomyosarcoma cells (RKI), PS porcine kidney cells (RKI), and LLC-MK2 African green monkey kidney cells (ATTC CCL-7, kindly provided by Lia van der Hoek, Academic Medical Center Amsterdam, The Netherlands).

Vero cells seemed to support virus growth efficiently but produced no CPE. Interestingly, there was a remarkable difference between Vero E6 and Vero FM cells (Table 1). In our hands these cells also showed differences in growth of SARS-CoV. Vero FM consistently showed more pronounced CPE than Vero E6 but there were no significant differences in RNA amplification (not shown).

**Figure 1:**
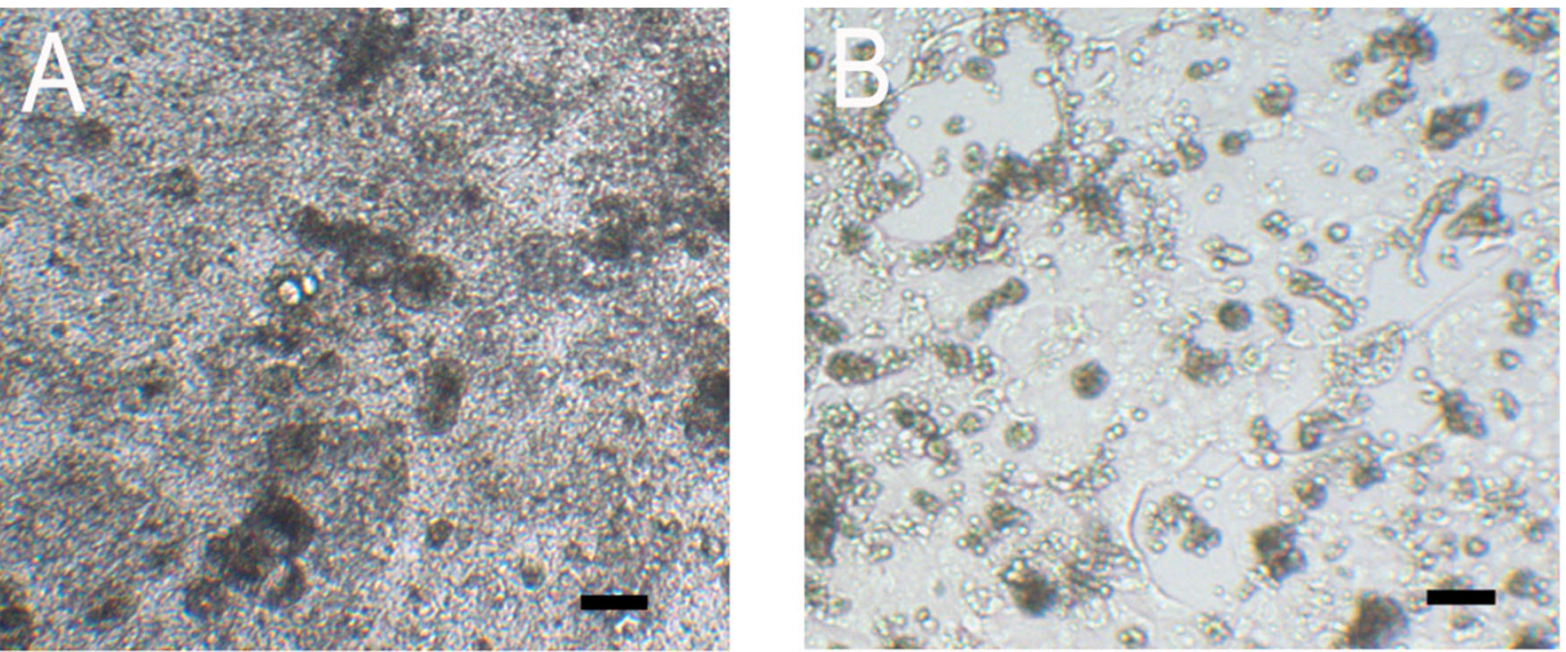
**Cytopathogenic effect of hCoV-NL63 on human colon carcinoma cells (CaCo-2)**. CaCo-2 cells 5 days after infection with hCoV-NL63 at an multiplicity of infection of 0.1 (agarose overlay technique). A, mock-infection; B, infection. Photographs were taken at 40-fold magnification; bars represent 20 μm.

CaCo-2 cells amplified virus RNA most efficiently, and showed a clearly visible CPE starting from day 4 after infection. Cells became rounded, detached from the surface, and showed morphological signs of cell death (Figure 1).

**Figure 2:**
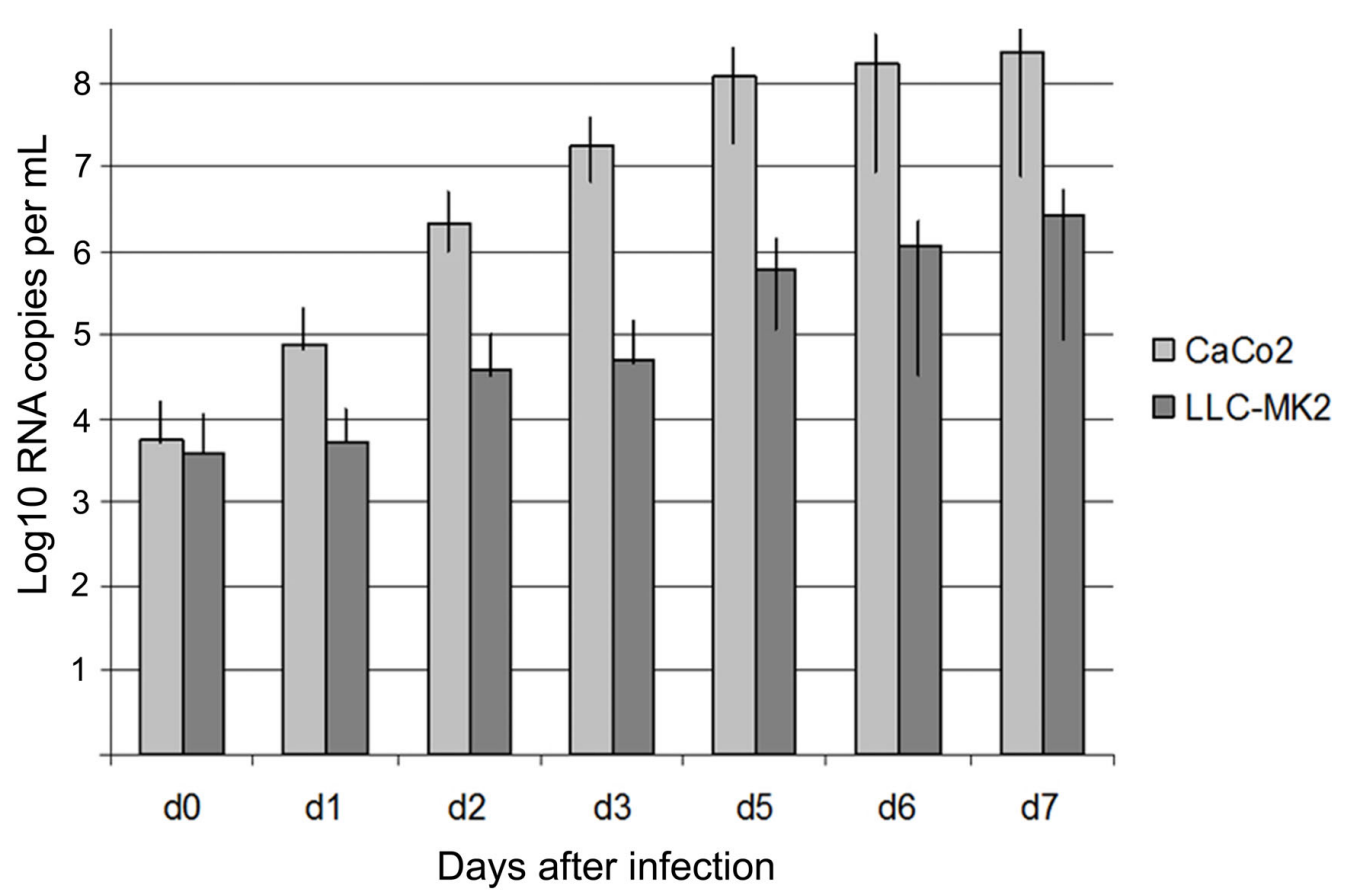
**Growth kinetics of hCoV-NL63 on LLC-MK2 and CaCo-2 cells**. 25 cm^2 ^flasks of LLC-MK2 or CaCo-2 cells were infected at multiplicities of infection of 0.005 for 1 h, washed twice with PBS, and subsequently supplied with 10 mL DMEM. Samples were taken daily from day 0 to 7 (except day 4) and analyzed by real time RT-PCR. Error bars indicate ranges of three independent experiments.

For confirmation of differential replication efficiencies, CaCo-2 and LLC-MK2 cells were infected in parallel. Both cell lines were seeded in 25 cm^2 ^flasks, and infected at multiplicities of infection of 0.005. Samples of supernatants were taken daily from day 0 to 7 and analyzed by real time RT-PCR. As shown in Figure 2, CaCo-2 cells replicated virus more efficiently than LLC-MK2. From day 3 onward, RNA concentrations were more than 100 fold higher in CaCo-2 cells. Because of the clear CPE observed in CaCo-2 cells, these cells were tested for their utility in a cytopathogenic plaque assay.

#### Comparison of different overlays

Three overlay techniques commonly used for plaque assays were tested for their suitability [23]. CaCo-2 cells were infected in 6-well plates with hCoV-NL63. After one hour, supernatants were removed, cells washed with PBS, and overlaid as follows.

For CMC overlays, 1 mL fresh DMEM was added to each well. Subsequently 1 mL of 1.6% CMC solution was slowly added per well. Agarose overlays (1% final concentration) were prepared by melting 2% agarose at 70°C, cooling it in a water bath to 42°C, and mixing it immediately before application with an equal volume of 2 × DMEM stored at room temperature. Two mL of the mixture were carefully applied to each well. Avicel overlays were made by mixing 2.4% Avicel solution with an equal volume of 2 × DMEM. 2 mL of the mixture were immediately added to each well.

**Figure 3:**
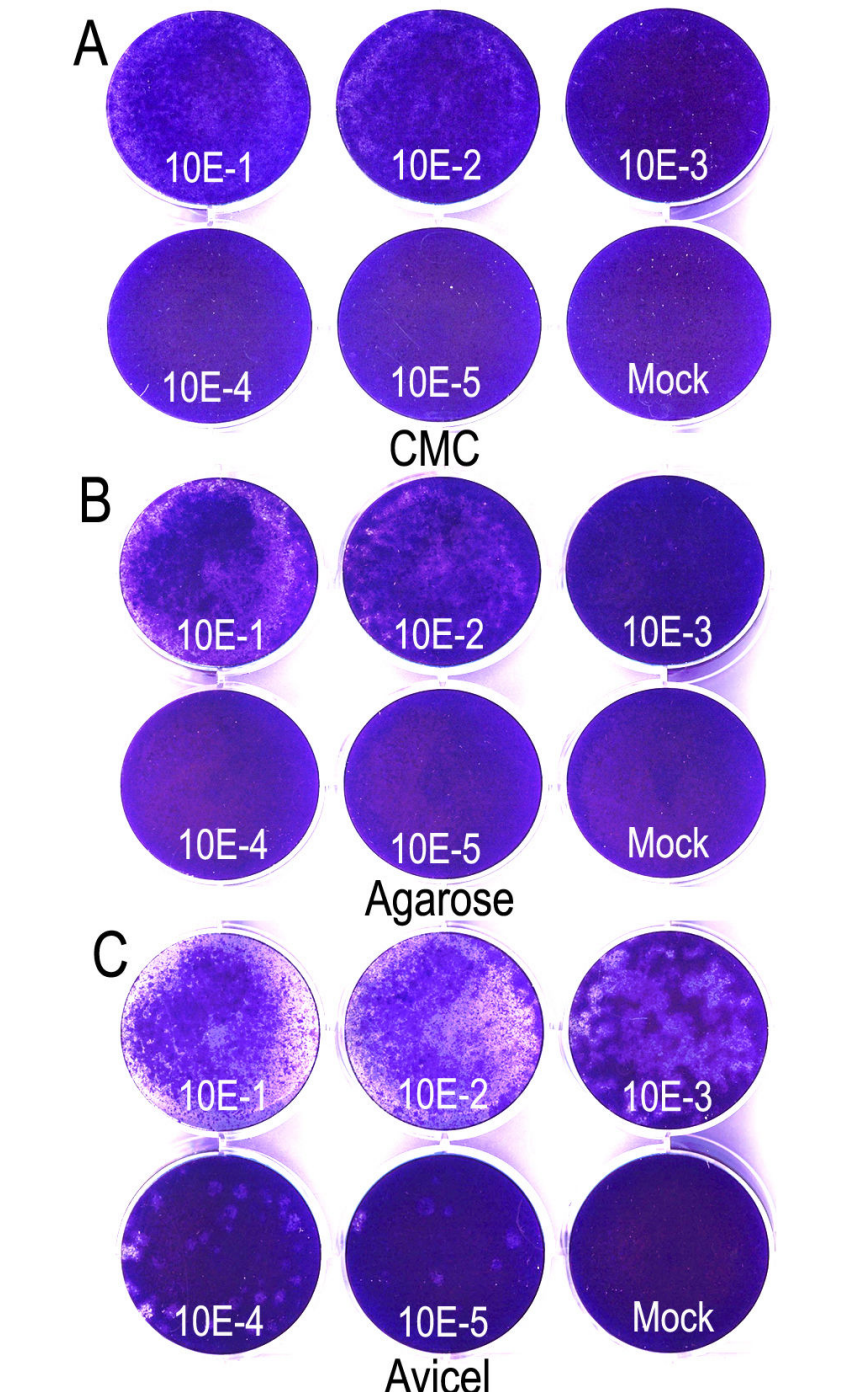
**Plaque assay for hCoV-NL63 on CaCo-2 cells using different overlays**. HCoV-NL63 was serially diluted on CaCo-2 cells (10e-1 until 10e-5). After 1 h of virus adsorbtion different overlays were added. After 5 days cells were fixed with 4% formaldehyde and stained with 0.2% crystal violet solution. A) carboxymethyl-cellulose; B) agarose; C) Avicel.

Plaque assays were incubated without disturbing at 37°C and 5% CO_2_. Overlays were removed on day five and cells were fixed with a solution of 4% formaldehyde in PBS. After 30 min the formaldehyde solution was removed, cells were washed twice with PBS, and stained with a 0.2% crystal violet solution. As shown in Figure 3, plaques were visible with all three overlays, but staining was clearest with Avicel.

#### Incubation times

**Figure 4:**
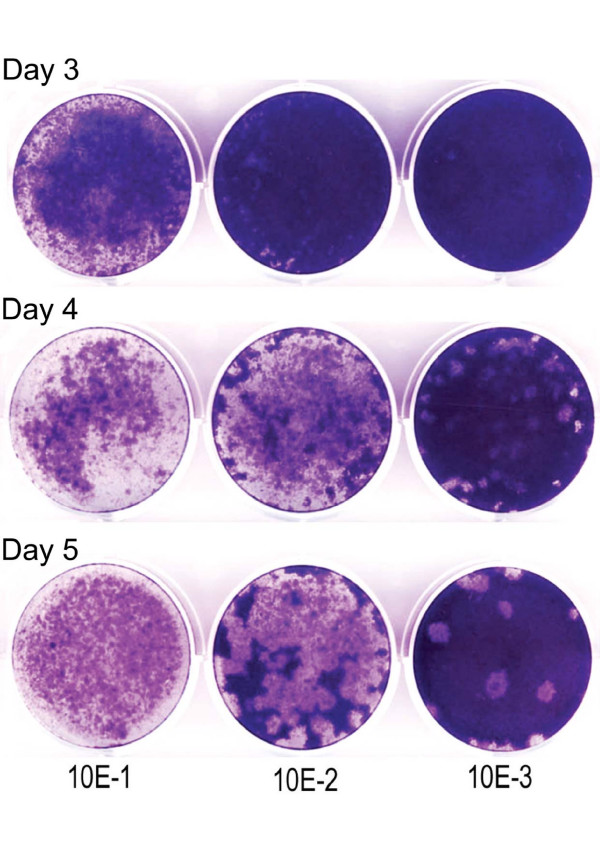
**Plaque assays with different incubation times**. Plaque assays were performed with Avicel overlay and incubated for 3, 4, and 5 days, respectively. The dilution factor of LLC-MK2 NP virus stock used for infection is shown on the bottom.

HCoV-NL63 culture with LLC-MK2 cells takes more than 7 days until first signs of weak CPE become visible. In order to test whether incubation times could be reduced with CaCo-2 cells, five plaque assays on virus dilution series were done with Avicel overlays and terminated by fixation after 1, 2, 3, 4, and 5 days, respectively. On days 1 and 2, no plaques were visible (not shown). Termination at day 3 yielded plaques only at high virus concentration (Figure 4). From day 4 onward, plaques were visible in the lowest detectable virus concentration. Plaques on day 5 were larger, but did not increase in number.

#### Plaque preparation

Work with hCoV-NL63 is complicated by low infectious titers in virus stock solutions. In order to obtain more infectious virus solutions, our standard virus stock LLC-MK2 NP (see Materials and Methods section) was plaque-purified using the agarose overlay. Because life staining of cells with neutral red solution was not successful on CaCo-2 cells (not shown), we used an alternative technique of plaque preparation.

Limiting dilution infections were done on 6-well plates. After 5 days, cytopathic foci were identified by scanning through the wells with an inverted microscope at low magnification, lighting through the clear agarose overlay. The positions of CPE foci were marked with a permanent marker (it was helpful to turn up the microscope light for this). The agarose overlay was penetrated with a pipette and 10 to 20 μl of fluid was aspirated underneath the overlay. This fluid was resuspended in 100 μl of Opti Pro serum-free medium, which served as the starting solution for a new limiting dilution infection series in the next 6-well plate plaque assay. Three rounds of purification were done. After the last round, aspirated fluid was inoculated in 5 mL of Opti Pro serum-free medium, which was then overlaid on confluent CaCo-2 cells in a 25 cm^2 ^flask for infection. After infection for one hour and washing, 5 mL DMEM were added and flasks were incubated at 37°C, 5% CO_2 _for four days. Stocks were harvested and stored as described for the original LLC-MK2 stock in the Materials and Methods section. The purified virus is hereafter referred to as CaCo-2 PP (for plaque-purified).

**Figure 5:**
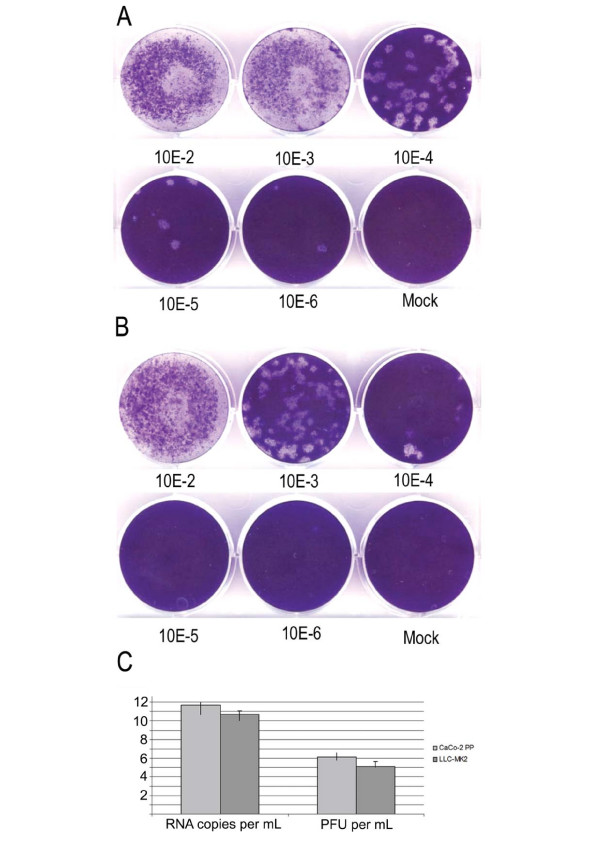
**Effect of plaque purification**. A, plaque assay with Avicel overlay on purified virus stock CaCo-2 PP. B, plaque assay on non-purified virus stock LLC-MK2 NP. C, viral RNA copies per mL of supernatant (left) and plaque forming units per mL of supernatant (right) for CaCo-2 PP and LLC-MK2 NP virus stocks (log scale). Error bars show ranges of three independent experiments.

To compare the infectivity of the plaque-purified virus with the original LLC-MK2 virus stock (see Materials and Methods section), viral titres were determined by Avicel plaque assay as shown in Figure 5. CaCo-2 PP was about 10-fold more infectious than LLC-MK2 NP. Plaque assays were repeated three times (not shown). Mean titres were determined to be 1.4 × 10e6 PFU/mL and 1.3 × 10e5 PFU/mL, respectively, for CaCo-2 PP and LLC-MK2 NP. Absolute quantification of virus RNA by real-time RT-PCR yielded 4.8 × 10e11 RNA copies/mL for CaCo-2 PP and 5.3 × 10e10 copies/mL for LLC-MK2 NP.

It was interesting to note that both virus stocks had rather high RNA concentrations as opposed to their infectivities. PFU/RNA ratios were 2.92 × 10e-6 for CaCo-2 PP and 2.45 × 10e-6 for LLC-MK2 NP. This high excess of RNA over infectious units might be attributable to the virus harvesting procedure, possibly releasing nonpackaged RNA along with virus particles during freeze-thawing. Because PFU/RNA ratios were very similar for both stocks, it appeared unlikely that elimination of defective interfering particles had contributed the gain of infectivity. It will be interesting in future studies to see whether hCoV-NL63 might have adapted to CaCo-2 cells during plaque purification.

## Conclusion

CaCo-2 cells seem to support hCoV-NL63 replication significantly better than hitherto used culture cells. Their application for a cytopathogenic plaque assay facilitates quantification of infectivity and enables studies using plaque morphology. Short incubation time of 4 days is compatible with high-throughput applications such as drug screening. The use of Avicel as an overlay is favourable for plaque quantification, whereas agarose overlays are preferred for plaque purification. Virus stock of increased infectivity will be beneficial for antiviral screening, animal modelling of disease, and other experimental tasks.

## Methods

### Cell cultures

All cells were cultivated in DMEM (Dulbecco's Modified Eagles Medium) (PAA, Cölbe, Germany) with 4.5 g/L Glucose (PAA), supplemented with 10% Foetal Bovine Serum "GOLD" (PAA), 1% Penicillin/Streptomycin 100 × concentrate (Penicillin 10000 U/mL, Streptomycin 10 mg/mL) (PAA), 1% L-Glutamine 200 mM, 1% Sodium Pyruvate 100 mM (PAA), 1% MEM nonessential amino acids (NEAA) 100 × concentrate (PAA). Utilized cell cultures are identified in Table 1. For passaging, cells were detached using trypsin-EDTA (PAA), except CaCo-2 cells. These were routinely subcultured by scraping and pipetting for mechanical re-suspension.

### HCoV-NL63 virus stock solution

An eighth passage virus stock of hCoV-NL63 was kindly provided by Lia van der Hoek, AMC Amsterdam. It was grown in LLC-MK2 cells in limiting dilution series, recovering it three times from the last well of a dilution series still showing diffuse CPE. Subconfluent LLC-MK2 monolayers were infected in 75 cm^2 ^flasks with virus supernatant from the last round of limiting dilution culture at a ratio of 1:100 (200 μl virus supernatant in 20 mL of fresh medium). This concentration was the highest virus dilution still infectious in this culture format. The flasks were incubated at 37°C, 5% CO_2_, and harvested on day four. For harvesting, flasks were frozen at -70°C and thawed. Cells and supernatant were centrifuged for 10 min at 5000 rpm. Cleared supernatant was aliquoted and stored at -70°C. This virus stock is hereafter referred to as LLC-MK2 NP (for non-purified).

### Infection of cells

Cells were seeded in 6-well plates at approximately 4 × 10e5 cells per well and incubated until the monolayer was 70–80% confluent. CaCo-2 cells were grown to 100% confluence. Prior to infection cells were washed with 1 × phosphate buffered saline (PBS). Virus inoculum in 900 μL GIBCO Opti Pro serum free medium (Invitrogen, Karlsruhe, Germany) plus 1% Penicillin/Streptomycin (PAA) and 1% L-Glutamine (PAA) was added to each well. Inoculum was removed after one hour of incubation. Cells were washed twice with 1 × PBS and supplemented with 2 mL DMEM per well.

### RNA extraction and real time RT-PCR

Viral RNA was extracted from cell culture supernatant with the QIAamp Viral RNA mini Kit (QIAGEN, Hilden, Germany). Real time RT-PCR for hCoV-NL63 with absolute virus RNA quantification was performed as described previously [38].

### Media and overlays for plaque assays

A 2.4% (w/v) suspension of Avicel RC-581 (FCM BioPolymer, Brussels, Belgium) was prepared in distilled water and autoclaved (20 min 121°C)[23]. A 2% (w/v) suspension of agarose (Plaque Agarose, Biozym, Hessisch Oldendorf, Germany) was prepared in distilled water and autoclaved. A 1.6% carboxymethyl cellulose (CMC) solution was prepared by autoclaving CMC powder (BDH, Poole, UK) with a magnetic stirrer. Autoclaved powder was hydrated in DMEM at 1.6% (w/v) and stirred overnight until homogenous.

Double concentrated Dulbecco's modified Eagle medium (DMEM) was prepared by mixing DMEM (PAA) with 9.48 g/L DMEM Powder (Biochrom, Berlin, Germany), supplemented with 20% Foetal Bovine Serum "GOLD" (PAA), 2% Penicillin/Streptomycin 100 × concentrate (Penicillin 10000 Units/mL, Streptomycin 10 mg/mL) (PAA), 2% L-Glutamine 200 mM, 2% Sodium Pyruvate 100 mM (PAA), 2% MEM NEAA 100 × concentrate (PAA). Medium was sterilized by filtration.

## Competing interests

The authors declare that they have no competing interests.

## Authors' contributions

PH performed the experiments and wrote the manuscript. CD coordinated the experiments and wrote the manuscript. MAM performed the experiments and wrote the manuscript.
